# Association of polymorphisms in inflammatory cytokines encoding genes with severe cases of influenza A/H1N1 and B in an Iranian population

**DOI:** 10.1186/s12985-019-1187-8

**Published:** 2019-06-13

**Authors:** Mohsen Keshavarz, Haideh Namdari, Mohammad Farahmand, Parvaneh Mehrbod, Talat Mokhtari-Azad, Farhad Rezaei

**Affiliations:** 1 Department of Virology, Faculty of Medicine, Iran University of Medical Sciences, Tehran, Iran; 2 The Persian Gulf Tropical Medicine Research Center, The Persian Gulf Biomedical Sciences Research Institute, Bushehr University of Medical Sciences, Bushehr, Iran; 3 Department of Medical Immunology, School of Medicine, Tehran University of Medical Sciences, Tehran, Iran; 4 Department of Virology, School of Public Health, National Influenza Center, Tehran University of Medical Sciences, Tehran, Iran; 5 Influenza and Other Respiratory Viruses Department, Pasteur Institute of Iran, Tehran, Iran

**Keywords:** Influenza, Cytokine, Inflammatory response, Single nucleotide polymorphism, Influenza-like illness

## Abstract

**Background:**

The increased levels of blood cytokines is the main immunopathological process that were attributed to severe clinical outcomes in cases of influenza A, influenza B and people with influenza-like illness (ILI). Functional genetic polymorphisms caused by single nucleotide polymorphisms (SNPs) in inflammatory cytokines genes can influence their functions either qualitatively or quantitatively, which is associated with the possibility of severe influenza infections. The aim of the present case-control study was to investigate the association of polymorphisms in inflammatory cytokines genes with influenza patients and ILI group in an Iranian population.

**Methods:**

Total number of 30 influenza B, 50 influenza A (H1N1) and 96 ILI inpatient individuals were confirmed by Real-time RT-PCR and HI assays. The genotype determination was assessed for defined SNPs in IL-1β, IL-17, IL-10 and IL-28 genes.

**Results:**

The frequencies of the IL-1β rs16944 (*P = 0.007*) and IL-17 rs2275913 (*P = 0.006*) genotypes were associated with severe influenza disease, while the frequencies of IL-10 rs1800872 and IL-28 rs8099917 were not associated with the disease (*P > 0.05*). Also, the absence of A allele in IL-17 rs2275913 SNP increased the risk of influenza A (H1N1) infection (*P = 0.008*).

**Conclusions:**

This study demonstrated that influenza A- (H1N1) and B-infected patients and also ILI controls have different profiles of immune parameters, and individuals carrying the specific cytokine-derived polymorphisms may show different immune responses towards severe outcome.

## Background

Influenza viruses are known as worldwide human and livestock pathogens which have caused serious respiratory diseases and deaths over the past century [1–3]. Influenza viruses have always had potential to cause widespread pandemics whenever several risk factors including live poultry market, climatic factors and most importantly susceptibility of type A to high diversity upon combination of different pathogenic hemagglutinins (HA) and neuraminidases (NA) proteins are available [4, 5]. Since H1N1 has been known as responsible for 2009 pandemic, upon evaluation of the 3672 confirmed cases of influenza A (H1N1) in Iran between 22 May and 21 December 2009, Gouya and colleagues reported that infections occurred in all age groups with and without any predisposing factors [6]. Further, according to the data obtained from www.who.int/flunet, and even in Iran [7], influenza B activity remained low, however, in East Asia high levels of influenza activity were reported which need more and more attentions.

Besides smoking, pregnancy and obesity risk factors, host immune conditions such as dysregulation in systemic anti- and pro-inflammatory responses also affect influenza primary infections. Although, the infected cells produce cytokines for the initiation of an immune response as well as controlling viral replication, influenza infection causes hypercytokinaemia which results in extra-respiratory tissue destruction following serious complications, disease development and death [8]. High levels of transcription, production and functional activity of inflammatory or anti-inflammatory cytokines sometimes originate from genetic variation such as single nucleotide polymorphisms (SNPs), altered coding region and promoter or regulatory region of cytokines genes. Further, SNPs in certain alleles of pro-inflammatory cytokines resulted in susceptibility to a wide range of infections [9]. Regarding these cytokines, endothelial cells secrete Interleukin-1β (IL-1β) through NLRP3 inflammasome mechanisms that contribute to flu-induced inflammation in lung cells [10]. One of the typical functional SNPs was found in IL-1B promoter, T-to-C transition at situation − 31 (rs16944). It is located in a TATA-box motif of IL-1B and affects the transcription activity of IL-1B through binding of multiple transcription factors [11]. It has been shown that an increase in IL-1β cytokine causes lung damage during infection by influenza A [12]. A study showed rs16944 polymorphism in IL-1β contribution to an increased risk of gastric cancer with proinflammatory phenotype in Caucasian carriers [12]. Another study highlighted the association of IgAN (IgA nephropathy; the most common form of primary glomerulonephritis) with upper respiratory tract infections [13].

Also, deficiency or variation of interleukin-17 (IL-17), a cytokine mainly secreted by Th17 cells, has caused increased susceptibility to the infection of extracellular pathogens [14]. Exclusively, the rs2275913 SNP that is located in promoter of the IL-17A gene is associated with a lot of diseases. It has been reported that the presence of A allele at the rs2275913 SNP increases the secretion of IL-17A [15, 16]. A meta-analysis study showed that IL-17A rs2275913 polymorphism is remarkably linked to the risk of many types of cancer [17]. A recent study showed that rs2275913 SNP of the IL-17 gene is related to acute bronchiolitis severity and this SNP could lead to variations in IL-17 expression [18].

One of the other most important responses to viral respiratory infections in airway cells is interferon secretion; however, there is little information about the polymorphisms associated with the gene IL-28 and the disease outcome [19]. The SNP polymorphisms located at rs8099917 site near the interleukin (IL) 28B gene as a type III interferon group is associated with spontaneous clearance of or a sustained virological response to interferon (IFN) α and ribavirin treatment in hepatitis C virus (HCV) patients [20–22]. The rs8099917 was also shown to be associated with natural clearance of HCV [23].

IL-10 is a key component of anti-inflammatory cytokine systems that regulates and suppresses the expression of pro-inflammatory cytokines by macrophages to limit the damage caused by viral and bacterial infections [24]. In this context, the frequencies of the certain SNPs in IL-10 alleles in Mexican patients were associated with susceptibility to severe disease, while other SNPs were associated with protection from severe disease [25].

The rs1800872 polymorphism that is located at the 5′-flanking region of IL-10 promoter is related to increased intensity of autoimmune and infectious diseases and control the transcription and expression of IL-10 [26, 27]. Schuurhof et al., showed that rs1800872 SNP enhanced resistance to severe Respiratory Syncytial Virus (RSV) infection and IL-10 balance could reduce the harmful effects of the immune system [28]. The IL-10 rs1800872 was shown to contribute to an increased risk for virus-induced encephalitis as well [29]. Therefore, evaluation of all these polymorphisms in relation to influenza disease is of great importance.

Viruses take advantage of SNPs-driven variations in host cytokines that alter the transcriptional activity of their genes as well as association with the possibility of a wide range of influenza viruses [30]. Another challenge is monitoring or classifying people with influenza-like illness [31] that frequently occur after influenza virus vaccination. The immunological response which is determined by blood cytokines are different in these cases [32]. Therefore, evaluation of the secreted cytokines and their SNP variants might be important. Recently study by Rogo and colleagues reported the effects of defined SNPs in IL-1β, IL-10, IL-17, and IL-28 in flu A/H3N2 in Iranian population that showed a higher risk of developing a severe infection [33]. However, to the best of our knowledge, the profile and the role of these certain SNPs have not been identified in cases of flu A/H1N1 and B infected Iranian patients. Regarding this, the present study was performed to investigate the SNPs in IL-1β, IL-10, IL-17, and IL-28 in severe cases of flu A/H1N1- and B-infected patients and compare the outcome with ILI cases in an Iranian population.

## Methods

### Study design

We performed a case–control study on the specimens obtained from the National Influenza Centre, Tehran University of Medical Sciences (TUMS) from February 2017 to March 2017. Total number of individuals who participated in the study include: 30 influenza B positive, 50 influenza A (H1N1) positive and 96 ILI cases. Patients with severe influenza infection were approved by a positive viral test and ILI subjects were negative in terms of influenza tests. All the tests were repeated twice. Influenza patients and ILI cases were defined with the symptoms: fever> 38 °C, sore throat, cough, rhinorrhea, dyspnea, headache, vomiting, thoracic pain and anorexia. Written consent was acquired from all contributors and approved by Science and Bioethics committee of Tehran University of Medical Sciences.

### Viral RNA extraction and molecular detection

Viral RNA was extracted from 200 μl fluid samples by High Pure Viral Nucleic Acid kit (Roche, Germany) according to the manufacturer’s manual. Then, Real-time RT-PCR was performed by RT-PCR kit (QIAGEN, Germany) using specific primers and probes in Stepone pulse instruments (Applied Biosystems, Foster City, CA) according to the protocol. The primer sequences used to identify subtypes of influenza A/H1N1 and B are listed in Table 1.

**Table 1:** Real-time PCR primers and probes specifications

Primers/Probes	Sequence (5′ > 3′)
Flu A Forward	GACAAAATAACATTCGAAGCAACTGG
Flu A Reverse	GGGAGGCTGGTGTTTATAGCACC
Flu A Probe^a^	GCATTCGCAA″t″GGAAAGAAATGCTGG
Flu B Forward	TCCTCAAYTCACTCTTCGAGCG
Flu B Reverse	CGGTGCTCTTGACCAAATTGG
Flu B Probe^b^	CCAATTCGAGCAGCTGAAACTGCGGTG

^a^TaqMan probes are labeled at 5′- end with FAM and at the 3′- end with BHQ1

^b^TaqMan probes are labeled at 5′- end with ROX and at 3′- end with BHQ1

### Genotyping of SNPs

DNA from each specimen (serum) was extracted using Roche DNA purification kit (Roche, Germany) according to the manufacturer’s instructions. After extraction, genotype determination was carried out for polymorphisms in IL-1b rs16944, IL-10 rs1800872, IL-17 rs2275913, and IL-28 rs8099917 using TaqMan commercial probes (Applied Biosystems, Foster City, CA). Genetic information related to the SNPs is provided in Table 2. For Real Time RT-PCR all reactions were carried out with 10 μl of TaqMan SNP genotyping master mix (Life Technology, Carlsbad, CA), 0.2 μl of probes, 4.8 μl distilled water, 5 μl of DNA samples. The cycling temperature was selected as follow: 95 °C for 10 min, 40 cycles of 95 °C for 15 s, and 60 °C for 1 min.

**Table 2:** The analyzed SNPs genetic data used in the study

Gene (SNP)	Target	Location	Alleles	Context sequence
rs16944	IL-1β	Chr2: 113594867	G/A	TACCTTGGGTGCTGTTCTCTGCCTC[G/A] GGAGCTCTCTGTCAATTGCAGGAGC
rs1800872	IL-10	Chr1: 206946407	T/G	CTTTCCAGAGACTGGCTTCCTACAG[T/G] ACAGGCGGGGTCACAGGATGTGTTC
rs2275913	IL-17	Chr 6: 52051033	A/G	TGCCCTTCCCATTTTCCTTCAGAAG[A/G] AGAGATTCTTCTATGACCTCATTGG
rs8099917	IL-28	Chr 19: 39743165	G/T	TTTTGTTTTCCTTTCTGTGAGCAAT[G/T] TCACCCAAATTGGAACCATGCTGTA

### Statistical analysis

Statistical analysis was performed using Software packages SPSS 19 (IBM, Chicago, IL) and concordance between the two tests was determined using Chi-square (x^2^) test. *: P < 0.05, **: P < 0.01 and ***: P < 0.001.

## Results

### Participants

A total of 50 influenza A (H1N1), 30 influenza B and 96 ILI were participated in this study. The mean age for influenza A, influenza B and ILI cases were 37.18 ± 21.49 (1–86), 47 ± 20.2 (4–76) and 48.6 ± 25.8 (1–90) years, respectively. In respect to age groups; 7 (14%) of influenza A (H1N1), 4 (13%) of influenza B groups and 16 (15%) of ILI cases were aged less than 20 years. About 34 (68%) of influenza A (H1N1), 15 (50%) of influenza B groups and 45 (46%) of ILI cases were aged 20–60 years and 9 (18%) of influenza A (H1N1), 11 (36%) of influenza B, and 33 (34%) of ILI were aged > 60 years. In influenza A patients 28 (56%) were male and 22 (44%) were female, in influenza B group 14 (46%) were male and 16 (53%) were female and in ILI group 42 (43%) and 52 (54%) were male and female, respectively (Table 3). Also, the frequency of clinical symptoms in influenza A (H1N1) and B groups are indicated in Table 4.

**Table 3:** Demographic information of participated patients in this study

Group	Age (%)	Sex (%)	Total (%)
Influenza A (H1N1)	<= 20: 7 (14) 21–60: 34 (68) > 60: 9 (18)	Male: 28 (56) Female: 22 (44)	50 (28.4)
Influenza B	<= 20: 4 (13.3) 21–60: 15 (50) > 60: 11 (36.7)	Male: 14 (46.7) Female: 16 (53.3)	30 (17)
ILI	<= 20: 16 (15.6) 21–60: 45 (46.9) > 60: 33 (34.4)	Male: 42 (44.7) Female: 52 (55.3)	93(54.5)
Total	<= 20: 26 (14.8) 21–60: 94 (53.4) > 60: 53 (30.1)	Male: 84 (48.3) Female: 90 (51.7)	174 (100)

**Table 4:** Clinical symptoms of influenza-infected patients

Groups	Fever	Sore throat	Cough	Rhinorrhea	Dyspnea	Nasal congestion	Thoracic pain	Headache	Anorexia	Vomiting
Flu A (H1N1)	50 (100%)	34 (68%)	44 (88%)	25 (50%)	25 (50%)	46 (92%)	18 (36%)	34 (68%)	17 (34%)	15 (30%)
Flu B	30 (100%)	21 (70%)	25 (83%)	13 (43%)	18 (60%)	27 (90%)	12 (40%)	23 (76%)	13 (43%)	7 (23%)

### Genotyping

The allele and genotype frequencies for SNPs in IL-1β, IL-10, IL-17 and IL-28, are shown in Table 5. The analysis of genotype variation in influenza A (H1N1), influenza B and ILI groups demonstrated that the frequencies of AA, GA, GG genotypes of IL-1β (*p = 0.008*) and IL17 (*p = 0.024*) could be associated with the risk of flu infections, while no significant association were found in the SNP genotypes of IL-10 and IL-28. The results showed that in comparison with ILI group, genotypes AA, GA, GG of IL-17; rs2275913 in influenza A (H1N1) were statistically significant and associated with the risk of influenza infection (*p = 0.007*) (Table 6). Also, in comparison with ILI group, the genotypes AA, GA, GG of IL-1β; rs16944 in influenza B group were statistically significant, which were associated with the risk of influenza infection (*p = 0.014*) (Table 7).

**Table 5:** Genotype frequencies of IL-β, IL17, IL10 and IL28 in influenza A (H1N1), influenza B and ILI groups

Gene and genotype	Flu A (H1N1) (%)	Flu B (%)	ILI (%)	Crude *p* value
IL-1β; rs16944				
AA	6(19.4)	1(3.2)	24(77.4)	
GA	44(31.9)	27(19.6)	67(48.6)	.008*
GG	0(0)	2(33.3)	4(66.7)	
IL-17; rs2275913				
AA	3(20)	3(20)	9(60)	
AG	16(20)	10(12.5)	54(67.5)	.024*
GG	30(32.5)	14(18.4)	32(42.1)	
IL-10; rs1800872				
GG	3(37.5)	1(3.2)	4(50)	
TG	44(29.5)	27(19.6)	77(51.7)	.944
TT	3(27.3)	2(33.3)	7(63.6)	
IL-28; rs8099917				
GG	2(33.3)	0	4(66.7)	
GT	28(31.1)	14	48(53.3)	.757
TT	20(25.3)	16	43(54.4)	

*p* value is significant

**Table 6:** Genotype and allele frequencies of IL-β, IL17, IL10 and IL28 in influenza A (H1N1) and ILI groups

Gene and genotype/ Allele	Flu A (H1N1) (%)		ILI (%)		Crude *p* value	OR (95% CI)	Adjusted *P* value
IL-1β; rs16944							
AA	6(20)		24(80)				
GA	44(39.6)		67(60.4)		.057		.185
GG	0(0)		4(100)				
GG vs. GA/AA	0(0)	50(35.5)	4(100)	91(64.5)	.299	.000 (.000- NA)	.999
AA vs. GG/GA	6(20)	44(38.3)	24 (80)	71(61.7)	.061	.295 (.103–.845)	.023
A	56 (56)		115 (60)		.456	1.205(.738–1.967)	
G	44 (44)		75 (39)				
IL-17;rs2275913							
AA	3(25)		9(75)				
AG	16(22.9)		54(77.1)		.007*		.011*
GG	30(48.4)		32(51.6)				
GG vs. GA/AA	30(48.4)	19(23.2)	32(51.6)	63(76.8)	.002*	3.072 (1.461–6.461)	.003*
AA vs. AG/GG	3(25)	46(34.8)	9(75)	86(65.2)	.491	.635 (.154–2.621)	.530
A	22		72		.008*	2.108(1.207–3.682)	
G	76		118				
IL-10; rs1800872							
GG	3(42.9)		4(57.1)		.851		
TG	44(36.4)		77(63.6)				.575
TT	3(30)		7(70)				
TT vs. GG/TG	3(30)	47(36.7)	7(70)	81(63.3)	.670	.774 (.183–3.277)	.728
GG vs. TG/TT	3(42.9)	47(35.9)	4(57.1)	84(64.1)	.704	.810 (.165–3.974)	.795
T	50		91		.785	1.071(.655–1.749)	
G	50		85				
IL-28; rs8099917							
GG	2(33.3)		4(66.7)				
GT	28(36.8)		48(63.2)		.863		.683
TT	20(31.7)		43(68.3)				
TT vs. GG/GT	20(31.7)	30(36.6)	43(68.3)	52(63.4)	.543	.658 (.316–1.372)	.264
GG vs. GT/TT	2(33.3)	48(34.5)	4(66.7)	91(65.5)	1.000	1.228 (.206–7.323)	.821
*T*	68		134		.656	1.126(.667–1.900)	
*G*	32		56				

*OR* Odds ratio, *CI* Confidence interval (calculated by logistic regression model)

*p* value is significant

**Table 7:** Genotype and allele frequencies of IL-β, IL17, IL10 and IL28 in influenza B and ILI groups

Gene and genotype/Allele	Flu B (%)		ILI (%)		Crude *p* value	OR (95%CI)	Adjusted *P* value
IL-1β; rs16944							
AA	1(4)		24(96)				
GA	27(28.7)		67(71.3)		.014*		.026*
GG	2(33.3)		4(66.7)				
GG vs. GA/AA	2(33.3)	28(23.5)	4(66.7)	91(76.5)	.629	1.549 (.266 9.007)	.626
AA vs. GG/GA	1(4)	29(29)	24(96)	71(71)	.009*	.101 (.013–.786)	.029
A	29		115		.096	1.639(.914–2.939)	
G	31		75				
IL-17; rs2275913							
AA	3(25)		9(75)		.176		.305
AG	10(15.6)		54(84.4)				
GG	14(30.4)		32(69.6)				
GG vs. GA/AA	14(30.4)	13(17.1)	32(69.6)	63(82.9)	.086	2.031 (.851 4.849)	.111
AA vs. AG/GG	3(25)	24(21.8)	9(75)	86(78.2)	.801	1.227 (.301–4.994)	.775
A	16		72		.264	1.449(.754–2.786)	
G	38		118				
IL-10; rs1800872							
GG	1(20)		4(80)		.871		.634
TG	28(26.7)		77(73.3)				
TT	1(12.5)		7(87.5)				
TT vs. GG/TG	1(12.5)	29(26.4)	7(87.5)	81(73.6)	.385	.391 (.046–3.353)	.391
GG vs. TG/TT	1(20)	29(25.7)	4(80)	84(74.3)	1.000	.631 (.065–6.127)	.691
T	30		91		.820	1.071(.596–1.924)	
G	30		85				
IL-28; rs8099917							
GG	0(0)		4(100)				.244
GT	14(22.6)		48(77.4)		.588		
TT	16(27.1)		43(72.9)				
TT vs. GG/GT	16(27.1)	14(21.2)	43(72.9)	52(78.8)	.440	1.314 (.5693.036)	.522
GG vs. GT/TT	4(100)	91(75.2)	0(0)	30(24.8)	.572	.000 (.000 - NA)	.999
T	46		134		.356	.728(.371–1.430)	
G	14		56				

*OR* Odds ratio, *CI* Confidence interval (calculated by logistic regression model)

*p* value is significant

The comparison of genotype variation in influenza groups (A and B) versus ILI group revealed that the genotypes AA, GA, GG of IL-1β; rs16944 and AA, GA, GG of IL-17; rs2275913 were statistically significant and associated with the risk of influenza infection, (*p = 0.007*) and (*p = 0.006*), respectively (Table 8). On the other hand, the frequency of A, G, and T alleles were evaluated in influenza A and B, and ILI groups. The results showed that the absence of alleles A in IL-17; rs2275913 SNP increases the risk of influenza A (H1N1) infection (*p = 0.008*) (Table 6).

**Table 8:** Genotype and allele frequencies of IL-β, IL17, IL10 and IL28 in influenza (A & B) and ILI groups

Gene and genotype	Flu A & B (%)		ILI (%)		Crude *p* value	OR (95% CI)	Adjusted *p* value
IL-1β; rs16944							
AA	7(22.6)		24(77.4)				
GA	71(51.4)		67(48.6)		.007*		
GG	2(33.3)		4(66.7)				.027*
GG vs. GA/AA	2(33.3)	78(46.2)	4(66.7)	91(53.8)	.689	.547 (.096–3.127)	
AA vs. GG/GA	7(22.6)	73(50.7)	24 (77.4)	71(49.3)	.004*	.235 (.091–.610)	
A	85		115		.163	1.353 (.884–2.070)	
G	75		75				
IL-17; rs2275913							
AA	6(40)		9(60)				
AG	26(32.5)		54(67.5)		.006*		.019*
GG	44(57.9)		32(42.1)				
GG vs. GA/AA	44(57.9)	32(33.7)	32(42.1)	63(66.3)	.002*	2.589 (1.3714.889)	
AA vs. AG/GG	6(40)	70(44.9)	9(60)	86(55.1)	.717	.810 (.266–2.467)	
A	38		72		.011*	1.831 (1.144–2.928)	
G	114		118				
IL-10; rs1800872							
GG	4(50)		4(50)		.760		
TG	72(48.3)		77(51.7)				
TT	4(36.4)		7(63.6)				.510
TT vs. GG/TG	4(36.4)	76(48.4)	7(63.6)	81(51.6)	.439	.619 (.170–2.256)	
GG vs. TG/TT	4(50)	76(47.5)	4(50)	84(52.5)	1.000	.731 (.168–3.178)	
G	80		85		.755	.934 (.609–1.433)	
T	80		91				
IL-28; rs8099917							
GG	2(33.3)		4(66.7)				
GT	42(46.7)		48(53.3)		.889		
TT	36(45.6)		43(54.4)				.784
TT vs. GG/GT	36(45.6)	44(45.8)	43(54.4)	52(54.2)	.972	.882 (.474–1.641)	
GG vs. GT/TT	2(33.3)	78(46.2)	4(66.7)	91(53.8)	.68	.669 (.116–3.841	
G	46		56		.882	1.036 (.652–1.646)	
T	114		134				

*OR* Odds ratio, *CI* Confidence interval (calculated by logistic regression model)

*p* value is significant

## Discussion

In this case-control study, samples were obtained from 50 influenza A (H1N1), 30 influenza B and 96 ILI patients. All the patients and ILI cases were confirmed using molecular and serological assays (Real-time RT-PCR and hemagglutination inhibition), respectively. The ILI patients were negative for the influenza viruses by these tests. They also had no history of vaccination and chronic diseases like chronic obstructive pulmonary disease (COPD).

The immune response to influenza virus infection is the production of inflammatory mediators like chemokines, proinflammatory and regulatory cytokines. This response contributes to the pathogenesis and severity of influenza infection. Sometimes a cytokine storm leads to the bronchiolitis and alveolar edema which are the characteristics of severe influenza outcome [34]. A study on pediatric outpatients infected with influenza, identified a proinflammatory cytokine profile that was associated with symptom severity [35]. It has been highlighted that specific cytokine and chemokine inhibitors may have the potential for severe influenza virus infection [36].

According to the pervious study, delivery of influenza viruses into the lung of hosts is associated with the production of pro-inflammatory cytokines and induction of pneumonia [37], hence it is concluded that increased cytokines production during viral infection plays an important role in the viral pathogenesis and disease development. Further, the presence of SNPs within the coding or promoter regions of cytokine genes can influence the degree of cytokine expression [38].

In this regard, some studies suggested that SNPs in the IL-10 and IL-1β genes might be associated with the severe outcome in influenza outbreak [39, 40]. Also, another study demonstrated that polymorphisms of L-1β, IL-10, IL-17, and IL-28 genes involved in the inflammatory process affect the outcome of disease caused by influenza A/H3N2 virus [33].

Previous studies on polymorphisms in interleukin genes have shown association with the susceptibility to flu infection due to their influence on cytokine production. Here, we evaluated the polymorphisms of several pro-inflammatory genes in severe influenza A (H1N1), B cases and ILI patients. Based on the WHO case definition, all flu positive cases in our study were already diagnosed and confirmed as severe acute respiratory infections (SARI) and required hospitalization (https://www.who.int/influenza/surveillance_monitoring/ili_sari_surveillance_case_definition/en/).

A study by Joel Martinez-Ocana et al. [25], demonstrated that IL-10 production in all viral-infected patients were elevated, and another study claimed that genotypes GG and TG of IL-10 (rs1800872) had significant association with the increased risk of severe influenza infection, however, in our study IL-10 SNPs in three patient groups did not show any association with viral infection or ILI (Tables 6, 7 and 8). It should be noted that, the type of selected SNP, time of sample collection, and nationality might affect the host responses and analysis results. In contrast, the frequencies of the IL-10 − 592C in Mexican patients during the influenza pandemic A(H1N1) pdm09 infection were associated with susceptibility to severe disease [25]. Addressing the nationality, a study showed that the IL-10; rs1800872 was associated with the increased risk of infection with influenza A/H3N2 virus in Iranian population [33]. However, this SNP in the current study on the Iranian patients with flu A and B and ILI was not associated with significant flu infection or ILI groups (Tables 5, 6, 7 and 8). It reveals that apart from host immune responses, the subtype of flu viruses may be involved in different genotype variation expression.

IL-28, a member of the IFN-γ family with antiviral properties in consistent with IL-10 induce survival signaling in host cells. Pervious study showed the transplant patients who carry minor-alleles in the IL-28B gene (rs8099917, TG or GG) have significantly higher rates of seroconversion following influenza vaccination [41]. Our data in Tables 6, 7 and 8 showed that at rs8099917, TT is the major-allele and TG or GG are minor-allele genotypes, hence patients with minor-allele genotypes of IL-28B may be strongly associated with the immunosuppressive responses and host protection. Therefore, similar to IL-10, in the present study, we found that IL-28; rs8099917 SNPs were not associated with the severe influenza infections (*P > 0.05*) (Tables 6, 7 and 8). In contrast, two previous studies demonstrated that IL-28; rs8099917 SNPs developed flu A (H3N2) and HCV infections in Iranian and Italian patients, respectively [33, 42].

Additionally, in the present study, we found that through comparison of SNPs frequency of flu A-, flu B- and flu A + B- infected patients with ILI group, the IL-1β and IL-17 genotypes (GG, GA and AA) might be associated with the severe influenza infection. Several studies have shown that children and mice infected with H1N1 influenza, presented more severe clinical manifestations and expressed high level of IL-1β cytokine compared to the control group with mild clinical manifestations [37, 43]. In our study, genotype AA of IL-1β; rs16944 was shown (Tables 6, 7 and 8) to have significant association with influenza severe infection. Indeed, genotype AA in IL-1β; rs16944 protects patients against influenza B infection. These results were inconsistent with two studies that showed the IL-1β polymorphism AA versus GG/AG enhanced susceptibility to invasive flu A (H3N2) and mild infection after solid organ transplantation, respectively [33, 44].

The IL-17 cytokine is known with diverse functions, which any deregulation in IL-17 production results in the host progression of cancers, inflammatory diseases, autoimmune disorders, and clearance of viral or microbial pathogens [45–47]. In respect to this context, besides SNP and IL types, addressing the type of alleles shows that the presence of G allele in IL-17; rs2275913 SNP increases the risk of influenza A (H1N1) infection (*P = 0.008*) (Table 5). In contrast, high frequency of these alleles in other evaluated SNPs, suggested that sometimes these variations may be associated with the regulation of the immune responses as well as protection against severe infection. These data suggest that patients carrying allele “A” at position IL-17; rs2275913 lack the ability to control their inflammatory response which results in ineffective immune T cells responses against flu viral infection. Furthermore, similar to the findings of two other studies, H1N1 pandemic influenza virus induced Th17-secreted IL-17, which mediated the evolution of H1N1 mild and severe diseases [48, 49]. This data demonstrated that flu viruses benefitted from IL-17 SNPs towards developing their infection and lung injury.

## Conclusions

In conclusion, understanding and evaluation of the patterns of cytokine co-variation especially in regards to different SNPs would provide novel insights into the pathophysiology and severity of the disease. The data obtained from the current study, showed that genotypes GG and AA of IL-17; rs2275913 and IL-1β; rs16944, respectively might be associated with severity of influenza A/H1N1 and B infections but IL-10; rs1800872 and IL-28; rs8099917 SNPs did not show any association with the risk of infection.

## Data Availability

All data in case of need are available.

## Abbreviations

HA: Hemagglutinin
HCV: Hepatitis C virus
IL-1β: Interleukin-1β
ILI: Influenza-like illness
NA: Neuraminidase
SNPs: Single nucleotide polymorphisms
